# Caffeine modifies blood glucose availability during prolonged low-intensity exercise in individuals with type-2 diabetes 

**Published:** 2014-06-30

**Authors:** Luiz Augusto da Silva, Leandro de Freitas, Thiago Emannuel Medeiros, Raul Osiecki, Renan Garcia Michel, André Luiz Snak, Carlos Ricardo Maneck Malfatti

**Affiliations:** 1 Midwest State University of Parana, Pharmaceutical Science Postgraduate Program, Guarapuava, PR, Brazil; 2 State University of Santa Catarina, Physical Education Postgraduate Program, Florianópolis, SC, Brazil; 3 Federal University of Parana, Department of Physical Education, Curitiba, PR, Brazil; 4 Campo Real College, Department of Biomedicine, Guarapuava, PR, Brazil; 5 Midwest State University of Parana, Department of Physiotherapy, Guarapuava, PR, Brazil

**Keywords:** Diabetes *mellitus*, caffeine, supplementary feeding, exercise

## Abstract

**Objective:**

The study investigated the effect of supplementation with maltodextrin (CHO) alone or associated to caffeine during exercise in T2DM subjects.

**Methods:**

Pilot study, using eight subjects with T2DM, aged 55±10 years, received CHO (1 g/kg) or caffeine (1.5 mg/kg) alone or associated before exercise protocol. The exercise was executed at 40% heart rate (HR) reserve for 40 min, with 10-min recovery. Blood pressure (BP) and perceived exertion scale (Borg) were checked every 2 min. Blood glucose (BG) was checked every 10 min. For statistical analysis, ANOVA test was used and the value was considered statistically significant at *p* <0.05.

**Results:**

The results showed that BP and HR did not change significantly among all treatments. Caffeine promoted a significant reduction in BG of 75 mg/dL (65%, *p* <0.05) during 40 min of exercise protocol compared to all groups.

**Conclusion:**

Supplementation with 1.5 mg/kg of caffeine reduces BG concentration during prolonged exercise in T2DM patients.

## Abstract (es)

**Objectivo:**

Investigar los efectos de la suplementación con maltodextrina (CHO) sólo o combinado con cafeína durante el ejercicio en sujetos con diabetes tipo 2 .

**Métodos:**

Estudio piloto que incluyó ocho sujetos con DM2, de 55±10 años, el CHO (1g/kg) o cafeína (1.5 mg/kg) sólo o combinado antes del protocolo de ejercicio. El ejercicio se realizó a 40% de la frecuencia cardiaca (FC). Reserva del corazón durante 40 min con 10 min de recuperación. La presión arterial (PA) y la escala de esfuerzo (Borg) fueron revisadas cada 2 min. La glucosa en sangre (GS) se comprobó cada 10 min. El análisis estadístico se realizó mediante ANOVA, y consideró significación estadística un valor de *p* <0.05.

**Resultados:**

Los resultados muestran que PA, FC y Borg no difirió significativamente entre los tratamientos. La cafeína promueve una reducción significativa en los niveles de glucosa en la sangre de 75 mg/dL (65%, *p* <0.05), durante un protocolo de ejercicio de 40 min en comparación con todos los grupos.

**Conclusiones:**

Suplementación con 1.5 mg/kg de cafeína redujo significativamente los niveles de GS durante el protocolo de ejercicio en pacientes con DM2 .

## Introduction

Diet and glycemic control during exercise is very important, preventing or delaying complications during and after exercise, like hyperglycemia or hypoglycemia, frequently detected in non-monitored diabetic patients. In addition, blood glucose (BG) control has a positive impact on long-term clinical outcomes in individuals with diabetes by delaying the onset and slowing the progression of serious diabetes-associated complications 1.

The postprandial BG response is related to overall glucose control 2 and it is significantly affected by the amount and type of carbohydrate (CHO) and the rate of carbohydrate digestion 3. Slower rates of CHO digestion and absorption after a low-glycemic index meal result in a smaller rise in postprandial BG and a smaller rise in insulin 4. Furthermore, diet modifications designed for people with diabetes may provide other benefits besides BG control in rest and during exercise 5. The observed improvements in exercise performance with CHO ingestion have been attributed to maintenance of plasma glucose and glycogen availability 6. Elevation of BG associated with supplementation is suggested to improve aerobic performance through reduction of muscle glycogen use or through the use of BG as a predominant fuel source as glycogen becomes depleted 7. A recent study characterized the better dose for CHO supplementation, improved glycogen availability in muscle and liver. The CHO supplementation before exercise increased muscle and hepatic glycogen storage after prolonged exercise only by 1.0 g/kg in liver and 2.0 g/kg in muscle without insulin and lipid oxidation alterations 8.

In diabetic patients, diet control and BG availability pre-exercise is very important, avoiding risk situations during exercise. Prior studies revealed that diet manipulation pre-exercise, like caffeine supplementation can be employed as an ergogenic agent for a wide range of exercise conditions 9 and it is often proposed that this effect is mediated by enhancing fat oxidation and decreasing CHO use in active muscles, but without mechanisms to support this theory. In fact, caffeine can directly antagonize adenosine receptors in many tissues, including tissues in the central nervous and cardiovascular systems and skeletal muscle and adipose tissue. This could result in a multitude of responses including adrenaline and noradrenalin secretion, altered blood flow, increased sympathetic nerve activity and blood pressure (BP) and increased BG and triglyceride mobilization 10. 

Awareness of the impact of caffeine on glucose availability in type 2 diabetes mellitus (T2DM) patients is rare and very limited. Thus this study was performed to verify BG availability and cardiovascular alterations during aerobic exercise in diabetic patients pre-exercise CHO associated with caffeine feeding. 

## Materials and Methods

### Subjects

The pilot study group consisted of eight T2DM patients; aged 55±10 years (see clinical conditions on Table 1). The subjects received maltodextrin (1 g/kg), caffeine (1.5 mg/kg), caffeine associated with maltodextrin (caffeine and maltodextrin drink) or placebo prior to testing. Informed consent was obtained for the study in accordance to Resolution of the National Council of Health, which was approved by the Ethics Committee of the Midwest State University of Parana (p.79538/2012).

**Table 1.**
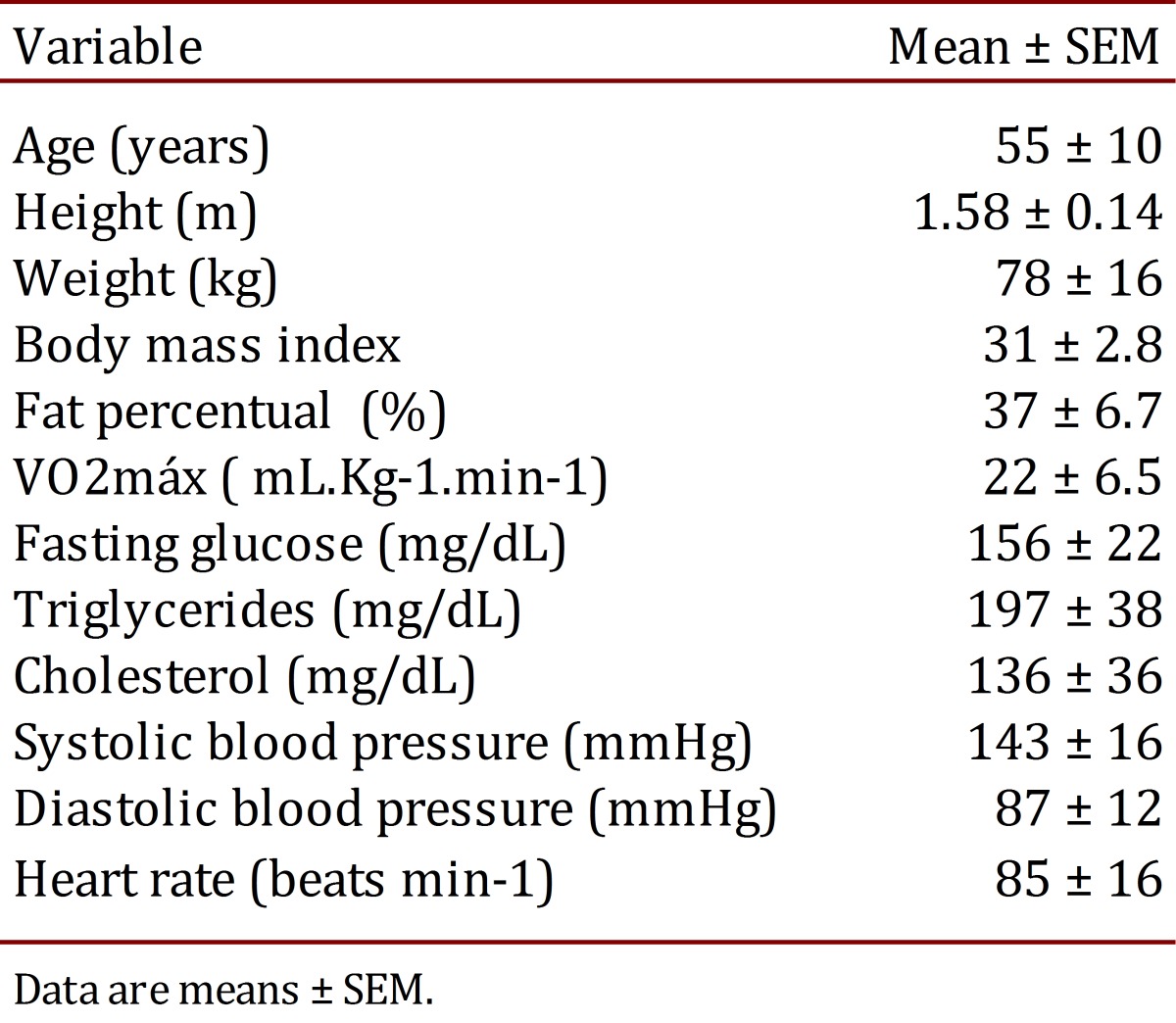
Clinical characteristics of subjects (n= 8)

### Inclusion and exclusion criteria 

 The study only included patients with medical diagnosis of T2DM in accordance with the Report by the Expert Committee on the Diagnosis and Classification of Diabetes mellitus 11, these subjects were free of other complications (cardiovascular disease, nephropathy, neuropathy, or retinopathy), as confirmed by their physicians. All subjects, but one, were considered non-caffeine users as defined by the consumption of <2 caffeinated coffee or tea beverages and/or <5 caffeine-containing soft drinks per week. Twelve hours prior to oral supplementation and exercise execution, hypertensive and diabetic medications were suppressed.

### Protocol of physical exercise

Indirect VO_2max_ was determined by using the Mile test protocol by Kline *et al* 12. The exercise protocol was performed by using a treadmill ergometer. Patients were asked to exercise at 40% heart rate (HR), reserve for 40 min, followed by a 10-min passive recovery rate (HRmax) for age (using tables from the American Heart Association). The exercise rate was adjusted for individuals to find intensity in 40% of HR reserve.

### Oral supplementation

The subjects received maltodextrin [1g/kg; Design Nutrition Advanced (DNA)] or caffeine (1.5 mg/kg; SIGMA reagents) or Maltodextrin associated with caffeine in the same prior doses or placebo (3.5 g of CHO; light tangerine juice) dissolved in distilled water 30 min before exercise. Patients were fasted 8 h before supplementations. In addition, they were instructed to follow a diet with no caffeine-containing products and alcohol, and avoid strenuous physical activity two days before the experiments.

### Laboratory analysis 

Prior to the prescription of exercise and treatments (one week before), the patients were evaluated in the laboratory, to obtain the biochemical, physiological, anthropometric and physical information. Body mass and height were measured by using anthropometrical devices (Welmy Corp., USA). Body fat was measured by means of the skinfolds technique 13 ^,^ 14 using a skinfold caliper (Cescorf Corp., USA). 

Cardiovascular parameters of BP (mercury column) and HR (Polar-T-61) were measured at rest, during different moments and after exercise session (checked every 2 min). The subjective perceived exertion and pain was rated by the Borg and Pain scale, respectively, used during exercise sessions 15 ^,^ 16.

Venous blood samples (5 mL) were drawn randomly for biochemistry laboratory at 9.00 a.m., and were centrifuged at 1,500 rpm during 8 min for plasma separation. Plasma triglycerides, cholesterol and glucose were dosed by glycerol phosphate oxidase/peroxidase and oxidase/peroxidase assay, respectively. In all methods, a BioSystems register kit was used.

Capillary blood samples (25 µL) were used to determine glucose concentrations during exercise during different moments (checked every 10 min) using a digital glucosimeter (ACCU - CHEK Performa*, *Roche^®)^.

### Statistical analyses

All data are expressed as means ± SEM. Statistical analysis was carried out by one-way analysis of variance (ANOVA) and *F*-values are presented only if *p* <0.05.* Post hoc* analysis was carried out, whenever appropriate, by the Tukey test.

## Results

 Within the exercise protocol, the patients were subjected to HR of predominant intensity at 40% of HR reserve. This intensity is in accordance with recent ADA recommendations 17 for diabetic patients. Table 2 shows a similar cardiac work in different phases during 40 min of aerobic exercise, followed for 10 min of recovery period, which did not show any significant difference among treatments. Hence, systolic and diastolic BP were not different during the same protocol. The present results can characterize a non-positive cardiovascular stimulus by caffeine or CHO use.

**Table 2.**
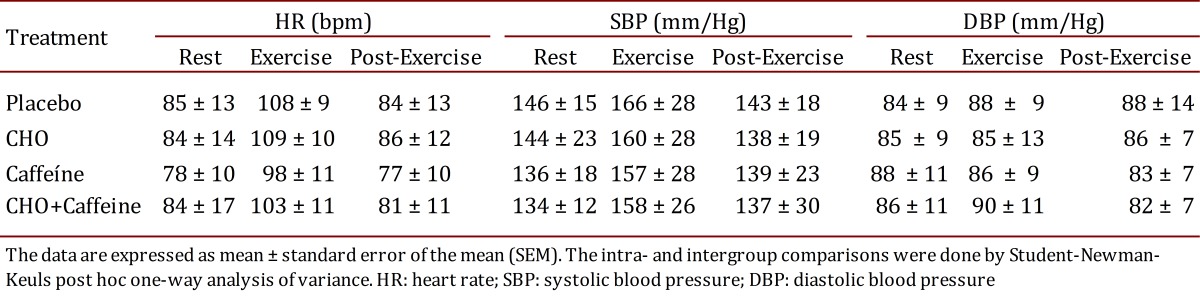
Cardiovascular responses before and after the respective supplementations before exercise (n= 08)

**Figure 1.**
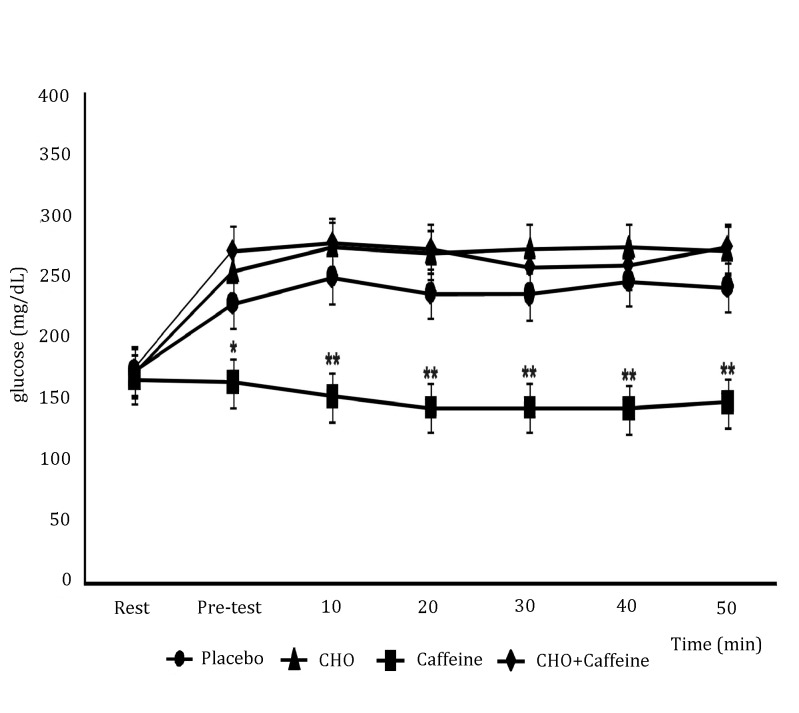
Concentration of blood glucose between treatments. Exercise was conducted based on a protocol of 40 min of exercise at 40% of HR reserve. Each data point shows the mean ± SEM (n= 8). * Statistical difference is shown between Caffeine group vs. CHO and CHO+Caffeine groups; ** Statistical difference is shown between Caffeine group vs. Placebo, CHO and CHO+Caffeine groups (*p* <0.05, one-way ANOVA with Tukey's post hoc tests).

Figure 1 shows results. Blood glucose was not different at rest before all supplementations. Although, after 30 min of supplementations, before exercise, caffeine-induced BG reductions compared to CHO + Caffeine (87%), CHO alone (83%) or placebo (65%) groups. In addition, BG was different during 40 min of exercise protocol, with caffeine-induced BG reductions compared to CHO + Caffeine (83%) and CHO alone (87%); [F(3,28): 5.3; p <0.001], but there was no significant difference between caffeine alone compared to placebo. Hence, BG was different during 10 min after the exercise protocol, with caffeine-induced BG reductions compared to CHO+Caffeine (85%), CHO alone (84%), or placebo (63%) [F_(3,28)_: 5.3; *p* <0.001].

## Discussion

Past and recent studies with caffeine-induced modifications in glucose homeostasis have been contradictory. However, most studies concerning acute caffeine response to glucose homeostasis revealed increased insulin resistance due to immediate secretion of epinephrine 18. In contrast, most long-term epidemiological studies revealed that long-term caffeine consumption via caffeinated drinks, such as coffee, had a rather beneficial action in glucose homeostasis to reduce the risk of T2DM 19. A recent study showed that in diabetic rats with insulin resistance and secretion deficit, long-term consumption of caffeine improved both sensitivity and glucose-stimulated insulin secretion, regardless of caffeine associated CHO addition; whereas, sucrose itself aggravated insulin resistance. Improved insulin sensitivity resulting from long-term caffeine consumption, in both normal and diabetic rats, was related to reduced body weight and fat 20. 

It has been reported that concentrations of caffeine could directly stimulate the â cell secretion of insulin 20 ^,^ 21. Caffeine alter**s** the expression of glucose transporter 2 (GLUT2) and glucokinase in â cells, involved in the phosphorylation of the glucose mechanism and, consequently, with the release of insulin 22. In the muscle, caffeine may improve the expression of GLUT4, by increasing concentrations of Ca^2+^ intracellular and also improved expression of AMPK enzyme 22. Caffeine could also be acting on adenosine receptors on the cell membrane of the hepatocyte, which are involved in glycogenolysis and gluconeogenesis 23.

Although this study was not designed to assess the mechanisms by which caffeine leads to glucose homeostasis, other studies showed increased serum insulin with the ingestion of caffeine 21 ^-^ 23. The results obtained by these studies, through measurement of C-peptide concentrations support the hypothesis that caffeine increases insulin concentrations via increased pancreatic secretion of the hormone rather than an alteration in insulin clearance 24 ^,^ 25, along with decrease BG concentrations by the association of caffeine with effect of better glucose uptake obtained with exercise 20 ^,^ 25.

Acute supplementation of caffeine and its effects can be seen from two different points: contribution to a suggestive increase of insulin release and BG control. However, it may have an adverse effect, it may have peaks of glucose uptake and cause hypoglycemia 20 ^-^ 22 ^,^ 24; whereas, the effects of administering caffeine on glucose homeostasis are not clear. In the present study, administering 1.5 mg/kg caffeine during 40 min of exercise contributed to maintenance of BG levels at ~140 mg/dL. It has been proposed that during exercise these diabetic patients need adequate BG maintenance at approximately 100 mg/dL 17 ^,^ 26.

Van Nieuwenhoven *et al*. 27, showed that consumption of 1.4 mg/kg of caffeine and 45 g of glucose administered for 90 min of exercise at 70% of maximal power output (Wmax) resulted in a significant increase of 23% in intestinal absorption of glucose consumption compared with glucose alone. According to the authors, caffeine could influence the activity of glucose transporters in the digestive system increasing BG. In the present study, intake of caffeine associated with CHO bear no difference in BG compared to intake of CHO alone, maybe due to the effect of caffeine on intestinal absorption of glucose, keeping BG to the Caffeine + CHO group the same as supplementation with CHO alone. Yeo *et al*. 28, demonstrated in their study that no difference in BG concentration was noted during exercise with carbohydrate intake associated to caffeine compared to treatment with carbohydrates. 

In relation to chronic use, Conde *et al*. 29, concluded that long-term caffeine intake (1 g/L) prevented development of insulin resistance by elevated blood insulin and lower blood glucose in diabetic rats.

A study by Noordzij *et al*. 30, revealed that the influence of caffeine on cardiovascular stimulation is directly related to the amount of caffeine consumed. The effect of caffeine on cardiovascular responses is due to the classic effect of caffeine on increased release of catecholamines, which directly influence the sympathetic nervous system and the consequent increase in blood pressure 31. In this study, no significant difference was observed among the groups possibly related to the dosage of 1.4 mg/kg of caffeine.

Thereby, the results showed in this study pilot, that acute caffeine ingestion in low doses prior to exercise could amplify the peripheral glucose uptake and consumption during aerobic exercise.
